# Epidrug-induced upregulation of functional somatostatin type 2 receptors in human pancreatic neuroendocrine tumor cells

**DOI:** 10.18632/oncotarget.9462

**Published:** 2016-05-19

**Authors:** Marije J. Veenstra, Peter M. van Koetsveld, Fadime Dogan, William E. Farrell, Richard A. Feelders, Steven W.J. Lamberts, Wouter W. de Herder, Giovanni Vitale, Leo J. Hofland

**Affiliations:** ^1^ Department of Internal Medicine, Division of Endocrinology, Erasmus MC, Rotterdam, The Netherlands; ^2^ Department Human Disease and Genomics Group, Institute of Science and Technology in Medicine, School of Medicine, Keele University, Keele, United Kingdom; ^3^ Laboratory of Endocrine and Metabolic Research, Istituto Auxologico Italiano IRCCS, Milan, Italy; ^4^ Department of Clinical Sciences and Community Health (DISCCO), University of Milan, Milan, Italy

**Keywords:** pancreatic neuroendocrine tumor, somatostatin receptor type 2, epigenetics, BON-1, QGP-1

## Abstract

Somatostatin receptors are a pivotal target for treatment of pancreatic neuroendocrine tumors (pNET), either with somatostatin analogues (SSA) or radiolabeled SSA. The highest affinity target for the most commonly used SSA is the somatostatin receptor type 2 (*sst*_*2*_). An important factor that may complicate treatment efficacy, is the variable number of receptors expressed on pNETs. Gene expression is subject to complex regulation, in which epigenetics has a central role. In this study we explored the possible role of epigenetic modifications in the variations in *sst*_*2*_ expression levels in two human pNET cell lines, BON-1 and QGP-1. We found upregulation of *sst*_*2*_ mRNA after treatment with the epidrugs 5-aza-2’-deoxycytidine (5-aza-dC) and valproic acid (VPA), an increased uptake of radiolabeled octreotide, as well as increased sensitivity to the SSA octreotide in functional cAMP inhibition. At epigenetic level we observed low methylation levels of the *sst*_*2*_ gene promoter region irrespective of expression. Activating histone mark H3K9Ac can be regulated with epidrug treatment, with an angle of effect corresponding to the effect on mRNA expression. Repressive histone mark H3K27me3 is not regulated by either 5-aza-dC or VPA. We conclude that epidrug treatment, in particular with combined 5-aza-dC and VPA treatment, might hold promise for improving and adding to current SSA treatment strategies of patients with pNETs.

## INTRODUCTION

Pancreatic neuroendocrine tumors (pNET) can be divided into two categories, e.g. functioning and nonfunctioning. Functioning tumors, e.g. insulinoma and glucagonoma, overexpress pancreatic hormones, which may lead to devastating paraneoplastic syndromes. Nonfunctioning tumors, which are the majority of pNETs, have neuroendocrine histology, however no hormonal syndrome associated with hypersecretion.

A primary target for medical treatment of pNETs is the somatostatin receptor (SSTR) type 2(*sst*_*2*_) [1]. The primary goal of *sst*_*2*_ targeted treatment is the control of excessive hormone secretion by functional tumors. Furthermore, the role of SSAs in the control and/or reduction of tumor progression has been studied in recent years. Two randomized double blind placebo controlled studies (PROMID and CLARINET) showed a growth reducing effect of both long acting octreotide and -lanreotide on midgut and pancreatic NETs [2, 3].

An additional important class of analogues is the radiolabeled SSAs. Radiolabeled SSAs are of significant importance for tumor imaging, localization and diagnosis [4]. In addition, they are used for peptide receptor radionuclide therapy (PRRT), an important treatment option for pNETs, particularly in inoperable and/or metastasized tumors [5]. Sufficient uptake levels of radiolabeled SSAs are essential and a higher uptake leads to a higher remission rate. As such, the number of SSTR expressed on the tumor cell may be important for the efficiency of treatment. High uptake on the pretreatment octreoscan is thus crucial for treatment outcome, next to high patient performance [5]. Moreover, it has been described that neoadjuvant treatment with PRRT could increase the number of patients eligible for surgery, as adjuvant treatment may decrease the chance of tumor spread following surgery or reduces growth of already present micrometastases [6].

*Sst*_*2*_ as being the SSTR to which octreotide and lanreotide bind with the highest affinity, makes this receptor the most important target receptor for treatment efficacy [7]. Okuwaki et al. showed the relationship between *sst*_*2*_ protein expression and survival in pNET patients [8]. This study also shows that *sst*_*2*_ expression is not present or not equally high in every pNET, limiting treatment options for patients with very low or no expression. To the best of our knowledge, no DNA mutations have been found in the *sst*_*2*_ gene that could account for low protein levels in a number of the patients.

Cells have various mechanisms to regulate gene expression, one of which is epigenetic regulation. Epigenetics comprises various modes of gene expression management that can interfere with transcription factors targeting gene promoters. Modifications to the epigenome, that include CpG island methylation and a wide range of histone modifications, impact on and can modulate gene expression. Alterations in the epigenetic regulatory system of a cell may also play a major role in cancer development and progression. Despite a gene being free of mutations, several epigenetic alterations can turn off tumor suppressor genes or turn on oncogenes, e.g. by chromatin condensation or hypermethylation of CpG islands in the promoter regions.

Because of the pliable nature of epigenetic modifications, it is possible to induce changes chemically. In recent years several drugs have been exploited that can reverse CpG methylation and others that inhibit histone de-acetylation. Two of these so-called epidrugs are the DNA methyltransferase inhibitor (DNMTi) 5-aza-2'-deoxycytidine (5-aza-dC) and histone deacetylase inhibitor (HDACi) valproic acid (VPA).

Torrisani et al. identified a novel transcription start site (tss) in *sst*_*2*_, upstream from the first described transcription start site by Petersenn et al. in 1999 [9, 10]. CpG methylation levels around the upstream tss are inversely associated with mRNA expression levels in several cell lines. In the investigated panel of cell lines it was found that epidrug treatment significantly increased *sst*_*2*_ expression in cell lines with low base line expression. Recently, Sun et al. described that VPA inhibited growth of BON-1 cells [11]. In their studies, VPA treatment induced cell cycle arrest as well as apoptosis. Furthermore, Greenblatt et al. previously showed that BON-1 cell xenografts in mice treated with VPA grow at a slower rate than in control mice [12].

In order to determine whether epigenetic mechanisms may be involved in the functional expression of *sst*_*2*_ in pNET cells, we studied two well characterized pNET cell line models, BON-1 and QGP-1 [13-15]. We present data on the expression and functionality of *sst*_*2*_ in untreated cells, as wells as in cells after treatment with the epidrugs 5-aza-dC and VPA. We studied whether epigenetic changes and regulation could be responsible for variations in *sst*_*2*_ expression and functionality. The effect of 5-aza-dC and VPA on *sst*_*2*_ expression, on uptake of radiolabeled *sst*_*2*_-preferring SSA, cAMP signaling, promoter CpG methylation, as well as enrichment of activating histone mark H3K9Ac and repressive histone mark H3K27me3 is reported. The ultimate future goal is being able to increase *sst*_*2*_ expression and/or signaling in pNET patients, in order to improve response to- and optimize the effect of SSA treatment and PRRT.

## RESULTS

### SSTR subtype mRNA expression

**Figure 1:**
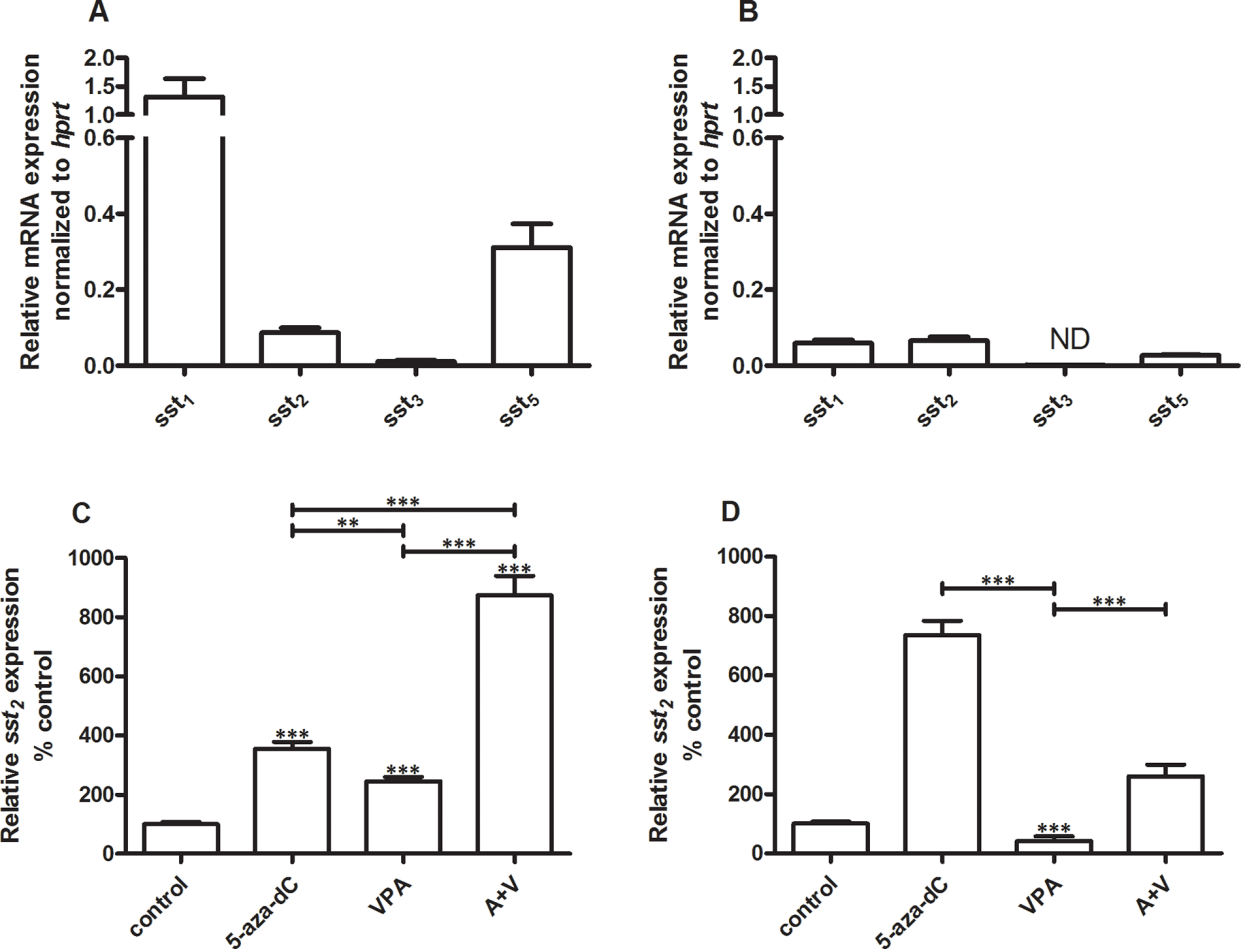
*Ssts* mRNA expression levels Mean expression levels ± SEM of *ssts* in BON-1 cells (n=2) **A** and QGP-1 cells (n=3) **B, C.** effect of treatment with 5-aza-dC (100 nM), VPA (2.5 mM) or the combination of both drugs on sst_2_ expression in BON-1 cells, **D.** effect of treatment with 5-aza-dC (50 nM), VPA (1 mM) or the combination of both drugs on sst_2_ expression in QGP-1 cells. Data are the mean ± SEM and expressed as the percentage of untreated control cells. The expression levels were normalized to housekeeping gene *hprt*. **p<0.01, ***p<0.001, ND: not detectable.

In BON-1 cells *sst*_*1*_ (1.3 ± 0.31; mean ± SEM) is expressed at the highest level relative to *sst*_*5*_ (0.31 ± 0.062) and *sst*_*2*_ (0.087 ± 0.013). *Sst*_*3*_ levels were very low (0.011 ± 0.0030) (Figure 1A). Compared to BON-1 cells, expression levels are generally low in QGP-1, with highest expression of *sst*_*2*_ (0.066 ± 0.0096), followed by *sst*_*1*_ (0.059 ± 0.0084) and *sst*_*5*_ (0.027 ± 0.0019). *Sst*_*3*_ expression is not detectable in QGP-1 cells (Figure 1B). Because in pNET treatment *sst*_*2*_ is the most important target receptor for SSAs like octreotide and lanreotide, we focused in the remaining part of our studies on this receptor subtype. For both cell lines EC_50_ concentrations for inhibition of cell growth were determined for 5-aza-dC and VPA. In BON-1 and QGP-1 EC_50_ values for 5-aza-dC were 100 nM and 50 nM, respectively. For VPA, the EC_50_ values were 2.5 mM in BON-1 and 1 mM in QGP-1 (data not shown). Cells were cultured in the absence or presence of EC_50_ concentrations of either 5-aza-dC, VPA or the combination of both drugs for 7 days. The two cell lines responded differentially to treatment with these drugs with respect to *sst*_*2*_ mRNA expression. In BON-1, 5-aza-dC and VPA alone significantly increase *sst*_*2*_ expression by 255 and 145 %, respectively. Furthermore, the combined treatment with 5-aza-dC and VPA enhanced expression by 770% (Figure 1C). In QGP-1 cells, 5-aza-dC alone stimulated *sst*_*2*_ expression by 635%, while VPA inhibited receptor expression by 60%. In addition, VPA also attenuated the stimulatory effect of 5-aza-dC in combined treatment to 160% stimulation (Figure 1D). *Sst*_*1*_, *sst*_*3*_ and *sst*_*5*_ mRNA expression results for epi-drug treatment in both cell lines can be found in the Supplementary Figure 1. For both cell lines, the changes in receptor expression of *sst*_*1*_, *sst*_*3*_ and *sst*_*5*_ with epidrug treatment, are minimal compared to the changes in *sst*_*2*_.

### Somatostatin receptor uptake

**Figure 2:**
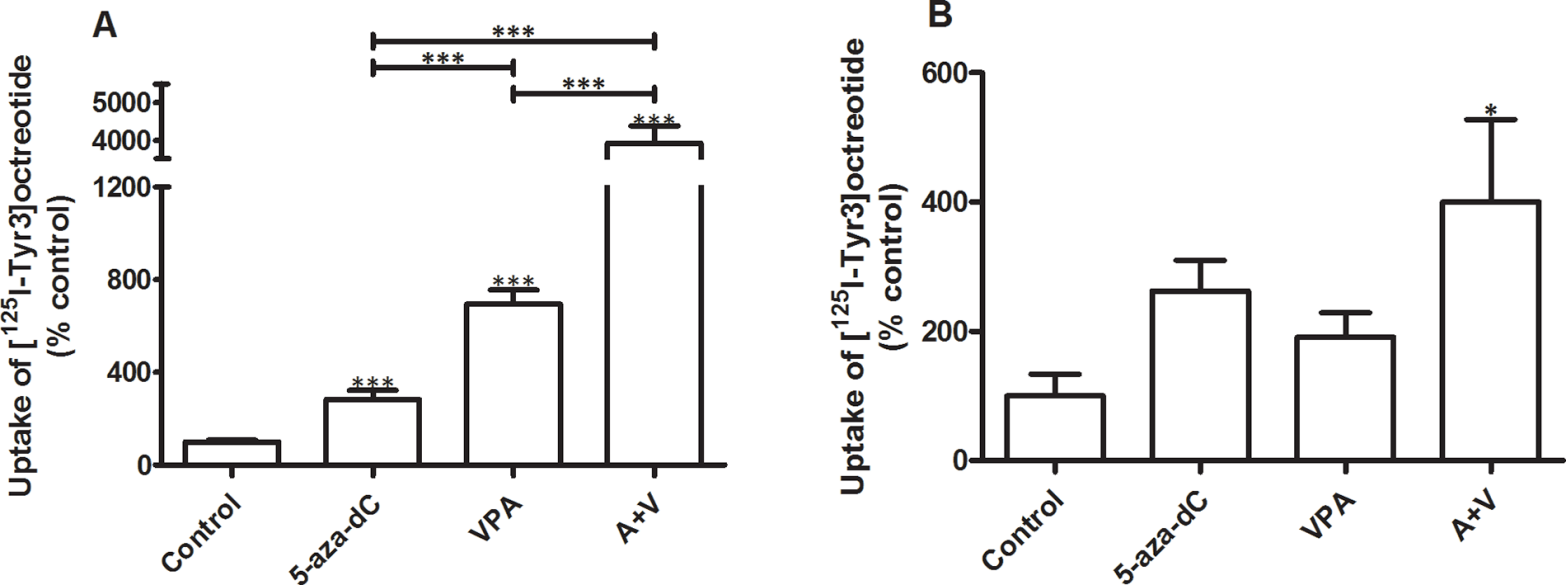
Uptake of radiolabeled [^125^I-Tyr^3^]octreotide Specific uptake of radiolabeled [^125^I-Tyr^3^]octreotide by BON-1 (n=3) and QGP-1 cells (n=3), without and with treatment with 5-aza-dC (BON-1: 100 nM, QGP-1: 50 nM), VPA (BON-1: 2.5 mM, QGP-1: 1 mM), or their combination. Specific uptake was calculated as the difference between total uptake and the uptake in the presence of excess unlabeled octreotide. Values were corrected for differences in cell number as measured by the DNA content per well. Values are expressed as the percentage of untreated control. **A.** BON-1, **B.** QGP-1. *p<0.05 ***p<0.001. Specific uptake values for control BON-1 cells amounted 0.14 ± 0.017% (mean ± SEM) and for QGP-1 0.11 ± 0.051% of added dose.

Figure 2 shows the effect of epidrug pretreatment on the uptake (internalization) of the *sst*_*2*_ preferring SSA [^125^I-Tyr^3^]octreotide. In the BON-1 cell line, 5-aza-dC increased uptake of [^125^I-Tyr^3^]octreotide by 185% and VPA by 595%. The combined treatment with EC_50_ concentrations of 5-aza-dC and VPA significantly increased the uptake of [^125^I-Tyr^3^]octreotide with 3820% (Figure 2A). In QGP-1 the effects of epidrug treatment are all stimulatory, although a statistically significant stimulation was observed only in cells that were treated with the combination of the drugs. Combined treatment with 5-aza-dC and VPA resulted in a statistically significant increased uptake of [^125^I-Tyr^3^]octreotide by 300% (Figure 2B).

### Effect on cAMP production

**Figure 3:**
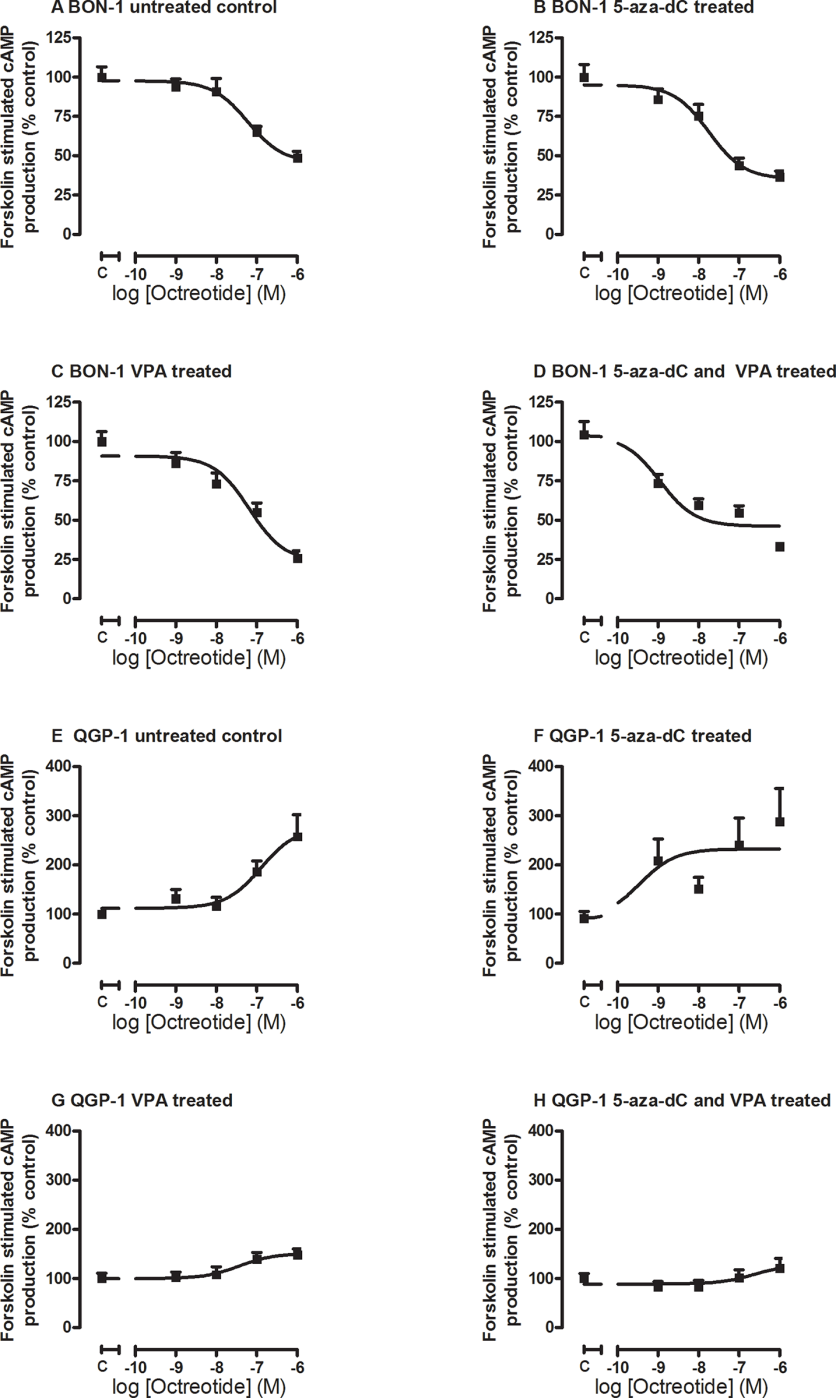
Octreotide-induced inhibition of forskolin-stimulated cAMP production Octreotide-induced inhibition of forskolin-stimulated cAMP production in BON-1 **A-D.** (n=3) and QGP-1 **E-H.** cells (n=3), without or with treatment with 5-aza-dC (BON-1: 100 nM, QGP-1: 50 nM), VPA (BON-1 2.5 mM, QGP-1: 1 mM), or their combination.

In order to evaluate whether epidrug treatment also could induce changes in the ability of the pNET cell lines to respond functionally to SSA treatment, we measured the effect of octreotide on forskolin-stimulated cAMP production in control and epidrug pretreated cells. Treatment of BON-1 cells with 5-aza-dC lowered the EC_50_ for the inhibitory effect of octreotide treatment on cAMP levels from 60 nM in untreated control cells to 18 nM in 5-aza-dC treated cells, indicating an enhanced effect of octreotide treatment at the level of cAMP inhibition (Figure 3A, 3B), although this difference did not reach statistical significance. VPA treatment did not explicitly influence the EC_50_ concentration (65 nM; Figure 3C), while the combined treatment further reduced the inhibitory EC_50_ concentration of octreotide significantly to 1 nM (p < 0.001 compared to control) (Figure 3D).

In QGP-1 cells, we found a reverse effect of octreotide on forskolin-stimulated cAMP production. The response to octreotide in the untreated QGP-1 cells did not show a dose dependent reduction in cAMP levels, but instead a stimulatory effect, with an EC_50_ of 130 nM in untreated cells (Figure 3E). The EC_50_ was reduced to only 0.36 nM in 5-aza-dC treated cells (Figure 3F), while VPA reduced EC_50_ to 37 nM and additionally lowered the maximal stimulation (Figure 3G). Combined treatment slightly increased the EC_50_ to 250 nM, and also lowered the maximal stimulation (Figure 3H). None reached statistical significance.

### DNA methylation

**Figure 4:**
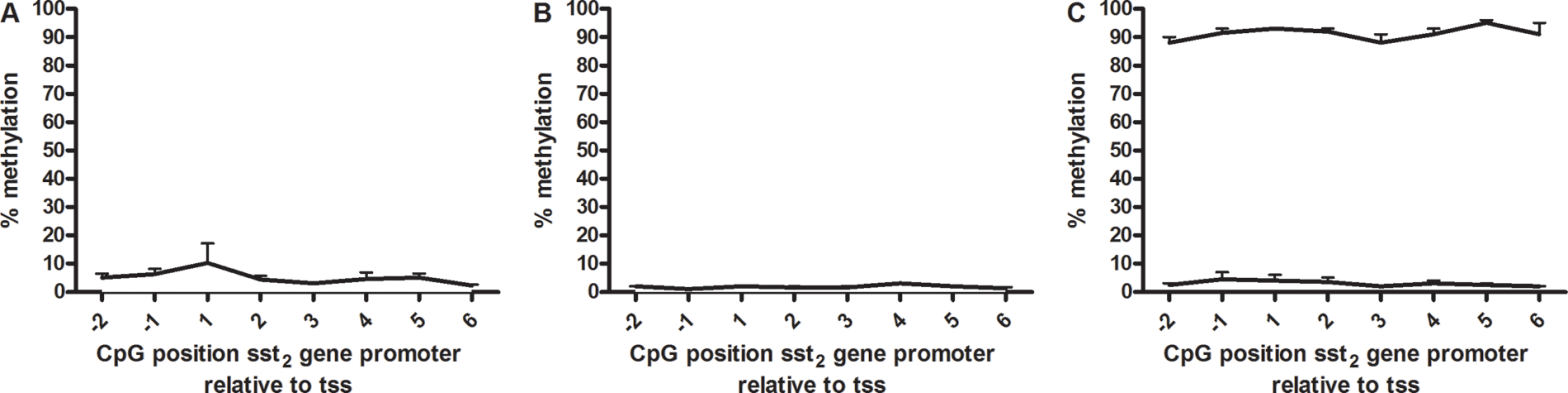
*Sst*_*2*_ promoter methylation Percentage *sst*_*2*_ promoter methylation of CpG positions surrounding the proximal transcription start site in **A.** BON-1 cells (n=3), **B.** QGP-1 cells (n=3) and **C.** high and low methylated control DNA (all mean ± SEM). The transcription start site is located between -1 and 1.

To determine the role of DNA methylation in *sst*_*2*_ regulation, methylation was quantitatively evaluated by pyrosequencing. The CpG sites studied were based on the work of Torrisani et al [9], who determined a number of CpG positions essential in the regulation of *sst*_*2*_ mRNA expression. These CpG positions are located in the promoter region, around the transcription start site. In both BON-1 cells and QGP-1 cells methylation levels were found to be low, all positions had under 10% methylation (Figure 4A and 4B). To verify the data, controls with high and low methylated DNA were sequenced in parallel as a positive and negative control. Figure 4C shows measured methylation levels between 85 and 96% and 2 and 7%, respectively, validating the pyrosequencing data in BON-1 and QGP-1 cells.

### Histone modification

To assess the role of the histone code in the regulation of *sst*_*2*_ expression, we evaluated the presence of two marks in untreated cells and the change of these marks under the influence of epi-drug treatment. By means of chromatin immunoprecipitation (ChIP) we determined enrichment of the promoter region of *sst*_*2*_ for active transcription mark H3K9Ac and for the repressive transcription mark H3K27me3.

**Figure 5:**
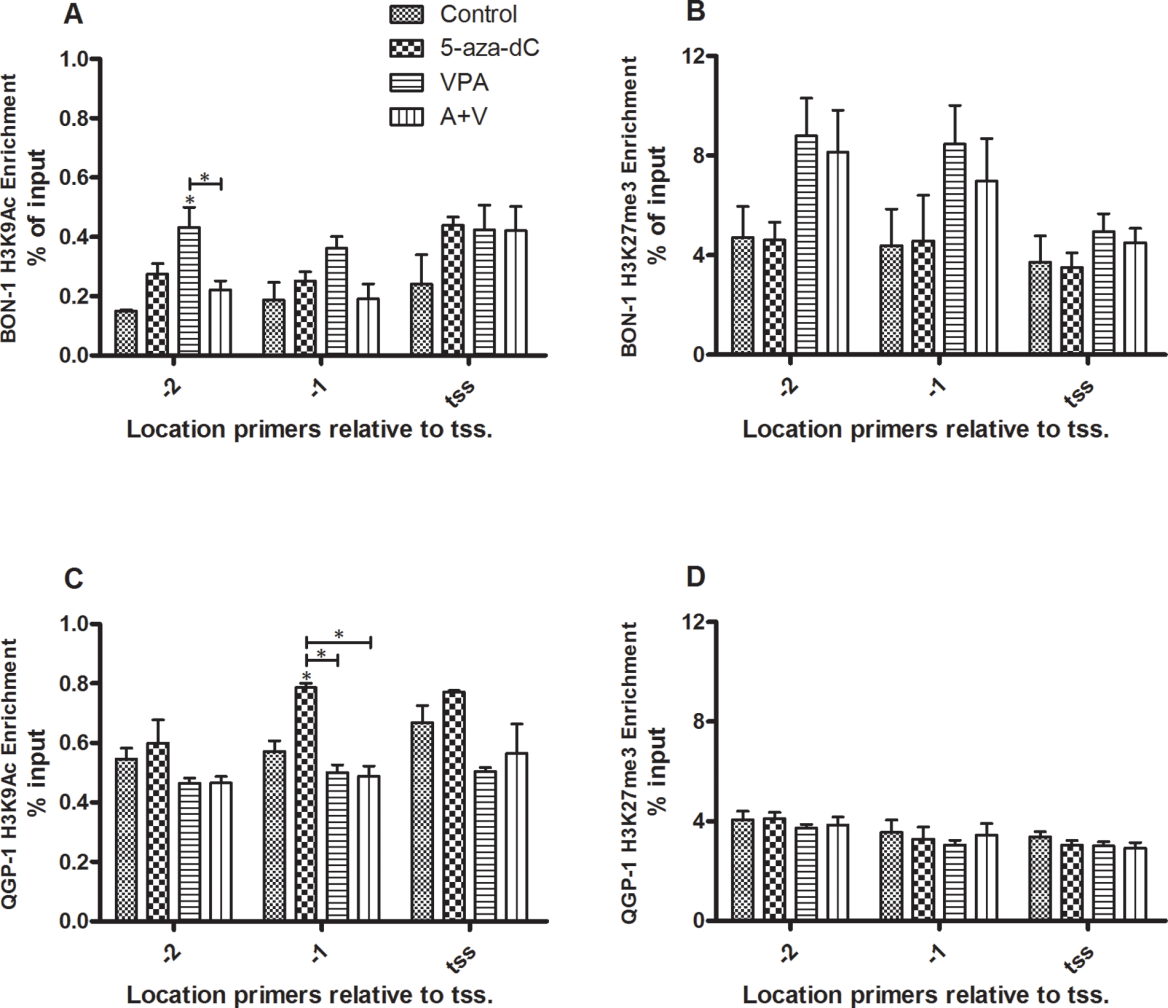
ChIP of histone marks H3K9Ac and H3K27me3 for the *sst*_*2*_ promoter Enrichment of the *sst*_*2*_ promoter as determined by ChIP for histone marks H3K9Ac and H3K27me3 (mean ± SEM) in BON-1 and QGP-1 cells, without or with treatment with 5-aza-dC (BON-1: 100 nM, QGP-1 50 nM), VPA (BON-1: 2.5 mM, QGP-1: 1 mM), or their combination. **A.** H3K9Ac in BON-1 cells (n=2) **B.** H3K27me3 in BON-1 cells (n=3) **C.** H3K9Ac in QGP-1 cells (n=2) **D.** H3K27me3 in QGP-1 cells (n=3). *p<0.05 vs untreated control.

Enrichment was determined at three positions in the *sst*_*2*_ promoter; at the transcription start site as described by Torrisani et al. and at two positions prior to this transcription start site [9]. Both modifications were detected in cell lines BON-1 and QGP-1. In BON-1 cells, individual 5-aza-dC and VPA treatment lead to a slight increase of activating histone mark H3K9Ac on all three positions, although this does not reach statistical significance, except for VPA treatment at position -2 (Figure 5A). The drugs combined gave no significant change (Figure 5A). No statistically significant effect of 5-aza-dC was observed on repressive histone mark H3K27me3 at either of the positions, while VPA showed a trend to increased enrichment at positions -2 and -1, which did not reach statistical significance, however (Figure 5B). H3K9Ac enrichment in QGP-1 cells is not influenced by VPA or combined treatment, while 5-aza-dC induces a statistically significant increase of the modification at position -1 relative to the tss (Figure 5C). Repressing histone mark H3K27me3 is in QGP-1 cells not influenced by either of the treatments relative to control (Figure 5D). In all experiments, efficiency was tested and confirmed with negative and positive control primers (data not shown).

## DISCUSSION

*Sst*_*2*_ is the highest affinity receptor for the conventional SSAs used in clinical setting, e.g. octreotide and lanreotide [7]. Expression of *sst*_*2*_ is highly variable between pNET patients [8, 16-19], where higher expression of *sst*_*2*_ is associated with better treatment results and better survival [5, 8]. The ability to upregulate *sst*_*2*_ expression in pNETs could improve treatment efficacy.

Epigenetic mechanisms play a key role in the regulation of gene expression [20]. DNMTs, HDACs and histone acetylases are some of the components involved in chromatin remodeling and, therefore, gene expression [21, 22]. We chose to work with two epidrugs, capable of altering epigenetic marks and which are already being used clinically; the DNMTi 5-aza-dC and the HDACi VPA. We determined the EC_50_ for both drugs in both cell lines, based on their inhibitory effect on cell growth. The determined concentrations are clinically feasible. Plasma concentrations that are reached for 5-aza-dC in hematological malignancies and solid tumors range from 0.04 μM to 5.6 μM and were reached using different treatment schedules [23, 24]. The EC_50_ concentrations of 100 nM in BON-1 cells and 50 nM in QGP-1 cells are within this range. The EC_50_ concentrations for VPA determined in both cell lines, 2.5 mM in BON-1 cells and 1 mM in QGP-1 cells, are close to therapeutically achievable serum concentrations, which range from 0.3 mM to 1 mM [25, 26].

The effect of 5-aza-dC and VPA on *sst*_*2*_ mRNA expression levels differed between the cell lines. In both cell lines 5-aza-dC stimulates expression, but in QGP-1 two times more efficiently. In contrast, VPA increases *sst*_*2*_ expression in BON-1, but is inhibitory in QGP-1. Moreover, while the effect of combined treatment leads to a considerably enhanced expression of *sst*_*2*_ in BON-1 cells, VPA negatively interferes with the stimulating effect of 5-aza-dC in QGP-1, as expected from the effect of VPA alone in this pNET cell line. The increase in *sst*_*2*_ expression induced by VPA treatment of BON-1 cells is in agreement with previous observations by Sun et al. [11].

In order to demonstrate the effect of epidrug treatment on the uptake of the radiolabeled *sst*_*2*_ preferring SSA octreotide, *sst*_*2*_ internalization studies were performed. In BON-1, 5-aza-dC and VPA treatment increased the uptake of [^125^I-Tyr^3^]octreotide significantly. Combined treatment significantly increases uptake by nearly 40-fold, consolidating the enhanced effect of both drugs seen on *sst*_*2*_ mRNA expression. Unlike the effect on mRNA expression, VPA stimulates uptake more than 5-aza-dC in BON-1.

While there was an inhibitory effect of VPA in QGP-1 on *sst*_*2*_ mRNA expression, uptake of [^125^I-Tyr^3^]octreotide was increased in VPA treated cells, as well as following 5-aza-dC treatment. Combined treatment induced a statistically significant increased uptake, which was higher than each compound individually, albeit not fully additive. Increased uptake does not necessarily need to be the result of increased numbers of *sst*_*2*_ on the cellular membrane. An increase in turnover and redirection of the receptor to the membrane might also cause a higher amount of internalized [^125^I-Tyr^3^]octreotide [27]. The results of the internalization studies show that, if these data can be extrapolated to the in vivo situation, both compounds, and particularly the combination, could have a potential role in improving treatment efficiency of PRRT in patients with pNETs.

To further confirm the functional outcome of the epi-drugs, we determined the effect of treatment on the efficacy of octreotide to inhibit cAMP production. Inhibition of cAMP is one of the main mechanisms via which *sst*_*2*_ activation results in inhibition of hormone release.

Octreotide inhibited forskolin-stimulated cAMP production in BON-1 cells with an EC_50_ of 60 nM, which was markedly decreased by 5-aza-dC treatment, to 18 nM. While VPA treatment had no distinct effect, leaving the EC_50_ at 65 nM, combined treatment enhances octreotide sensitivity even more than 5-aza-dC alone, to an effective EC_50_ dose of 1 nM. These data suggest a shift in the potency of octreotide to inhibit cAMP production to a more *sst*_*2*_-like (IC_50_ 0.38-0.60 nM) binding profile [7]. These data show that 5-aza-dC could play a major role in increasing sensitivity to treatment with *sst*_*2*_-preferring SSA, most likely by inducing an upregulation of *sst*_*2*_, and even more when combined with VPA. In untreated QGP-1 cells, octreotide treatment does not lead to inhibition of cAMP production, but instead increases cAMP expression with an EC_50_ of 130 nM. Pre-treatment with 5-aza-dC considerably reduces the EC_50_ dose to 0.36 nM, and although the angle of the effect remains an increase of cAMP production, 5-aza-dC did reduce the EC_50_ of octreotide to a clinically relevant concentration. Also VPA treatment and combined treatment increased cAMP production, but in line with the reduced *sst*_*2*_-expression, VPA alone, as well as combined VPA/5-aza-dC treatment lowered the maximal response to octreotide. To the best of our knowledge increased cAMP production upon SSTR activation has been previously described only in porcine somatotroph cells [28]. Currently, it is unclear whether this process can be found in tumors of patients, and whether this could play a role in sensitivity to treatment. This remains to be investigated.

Considering the variable *sst*_*2*_ expression levels in pNETs [8, 16-19], and the absence of known mutations, epigenetic modifications of *sst*_*2*_ regulatory elements might explain these differences between patients. By pyrosequencing we analyzed 8 CpGs present in the promoter region of the *sst*_*2*_ gene, which previously have been shown to be regulating *sst*_*2*_ expression [9]. In both BON-1 and QGP-1 low methylation levels were detected, around only 3% in BON-1 and 2% in QGP-1. These low levels suggest that CpG hypermethylation in this region does not play a role in low *sst*_*2*_ expression or loss of expression, although a role of CpG methylation in other regions of the *sst*_*2*_ gene cannot be excluded.

Epigenetics, however, comprise a wide range of modifications, such as a large series of histone modifications, controlling chromatin accessibility. Two well-known histone marks, H3K27me3, associated with transcriptional repression, and H3K9Ac, associated with active gene transcription were studied. In BON-1, both 5-aza-dC and VPA induced a slight increase in enrichment of active transcription mark H3K9Ac at all three studied positions, although this effect was only significant for VPA treatment at position -2. The general direction of the effect of treatment with 5-aza-dC and VPA on *sst*_*2*_ mRNA expression is reflected in the effect on enrichment of histone marks H3K9Ac in BON-1 cells. The effect on H3K27me3 is contradictory, as the expected effect is downregulation, whereas an (non-significant) upregulation is seen after treatment with VPA and the combination. Similar observations were made in QGP-1 cells. The absence of an effect of 5-aza-dC on H3K27me3, in combination with an increase in H3K9Ac in QGP-1, although not significant, is more in line with the observed increased *sst*_*2*_ expression. In conclusion, H3K27me3 appears thus to be a histone mark that does not play a major role in regulation of the *sst*_*2*_ promoter, while H3K9Ac does seem to play a role in activation of *sst*_*2*_ and is a good candidate for further studies.

As the treatment with epidrugs in our models is not selective in targeting genes, it cannot be excluded that indirect regulation of *sst*_*2*_ expression takes place. Transcription factors and other regulating factors might be upregulated or redirected, leading to increased mRNA expression. In addition, second messenger pathway participants might be influenced, or recycling of the receptor could be slowed, leading to enhanced functional response.

Currently, epidrugs have limited use in cancer treatment. Based on the results of this study, and the clinical trials performed so far with either 5-aza-dC or VPA or their combination [29, 30], these epidrugs alone or in combination, and with concomitant treatment with SSA, may be a future treatment approach in pNET patients that are otherwise not responding to- or suitable for any treatment.

In conclusion, the results of our study show that epidrug treatment, in particular with 5-aza-dC and VPA, might hold promise for improving current treatment strategies for patients with pNET with different types of SSA, in particular in patients with difficult to treat pNETs with low *sst*_*2*_ numbers. However, further experiments are needed to validate whether a similar upregulation of *sst*_*2*_ expression and *sst*_*2*_ functionality can be achieved in vivo.

## MATERIALS AND METHODS

### Cell culture

In this study two human pNET cell lines were used, BON-1 [13] and QGP-1 [14]. BON-1 cells were a kind gift from Dr. Townsend (University of Texas Medical branch, Galveston, USA). QGP-1 cells were purchased from the Japanese Collection of Research Bioresources Cell Bank (JRCB, Osaka, Japan). Both cell lines were confirmed as mycoplasm-free. Using short tandem repeat profiling the identity of the BON-1 and QGP-1 cell lines was confirmed [15]. BON-1 cells were cultured in DMEM-F12 supplemented with FCS (10% v/v) L-glutamine (2 mmol/L), fungizone (0.5 mg/L) and penicillin (1 × 10^5^ u/L). QGP-1 cells were cultured in RPMI-1640 supplemented with FCS (10% v/v) and penicillin (1 × 10^5^ u/L). Cells were cultured in 75 cm^2^ culture flasks (Corning Life Sciences B.V., Amsterdam, the Netherlands) at 37°C in a 5% CO_2_ humidified incubator and passaged by trypsinization every 7 days, with medium supplementation on the fourth day, until a maximum of 20 passages. For experiments cells were harvested with trypsin (0.05%)-EDTA (0.53 mM) and plated in 12 or 24 well plates (Greiner Bio-one B.V., Alphen aan den Rijn, the Netherlands). All media and supplements were obtained from Gibco (Life Technologies, Bleiswijk, the Netherlands) except for penicillin (Astellas Pharma B.V. Leiden, the Netherlands).

### Compounds

VPA sodium salt and 5-aza-dC powder were purchased from Sigma-Aldrich (Zwijndrecht, the Netherlands). VPA sodium salt was stored at -20°C and freshly prepared by dissolving in culture medium prior to each experiment and compound refreshing. 5-aza-dC was dissolved in H_2_O at a concentration of 10^-3^ M, aliquoted and stored at -20°C.

### Cell proliferation assay

To determine the optimal cell number for the culture of the cell lines in 24 well plates and determination of the EC_50_ concentrations for 5-aza-dC and VPA treatment, DNA measurement (as a measure of cell number) was performed. Cells were cultured and, in case of EC_50_ determination, treated in 24 well plates. Medium and compounds were refreshed after three days. After 7 days of treatment the medium was removed and cells were lysed with 150 μl ammonia solution (1 mol/L)-Triton-X100 (0.2% v/v) per well and incubated 10 minutes at 4°C. Plates were sonicated 5 seconds per well at an amplitude of 1400 microns to shear the DNA, and incubated for another 10 minutes at 4°C. Per well 1 ml assaybuffer (100 mmol/L NaCl, 10 mmol/L EDTA, 10 mmol/L Tris; pH 7.0) was added. Per well 20 μl was pipetted into a cell star plate, 96 wells (Greiner), along with a standard curve of calf thymus DNA (Sigma) and 200 μl Hoechst 33258 (Sigma-Aldrich). Measurement was performed at 350 and 455 nm excitation and emission wavelengths respectively on a Victor X4 plate reader (Perkin Elmer, Waltham, Massachusetts, USA) and counts were referenced against the standard curve.

### Quantitative RT-PCR

Quantitative RT-PCR was performed according to a method previously described [31, 32]. In brief, poly A+ mRNA was isolated from cells using Dynabeads oligo(dT)_25_ (Invitrogen Dynal AS, Oslo, Norway). The mRNA was eluted from the beads with 2 times 23 μl H2O incubated 2 minutes at 65°C. Subsequently cDNA was synthesized with twice 20 μl, once with Reverse Transcriptase and once without. The cDNA is diluted five times, Q-PCR was done on 5 μl sample with Taqman^®^ Universal PCR mastermix (Applied Biosystems, Life Technologies) and detection using an ABI Prism 7900ht sequence detection system (Applied Biosystems). Primer and probe sequences are shown in Table 1.

**Table 1:** Primer-probe sequences for Q-RT-PCR, PCR and sequencing primer sequences for pyrosequencing (Btn: biotin) and qPCR primer sequences for ChIP.

cDNA primers	Sequence
*hprt*	
Forward	CAC TGG CAA AAC AAT GCA GAC T
Reverse	GTC TGG CTT ATA TCC AAC ACT TCG T
Probe	CAA GCT TGC GAC CTT GAC CAT CTT TGG A
*sst*_*1*_	
Forward	CAC CGT GGC CAA GGT AGT AAA
Reverse	CCA CGA TGG GCA GGA TGA
Probe	CTG GGC GTG TGG GTG CTA TCG C
*sst*_*2*_	
Forward	TCG GCC AAG TGG AGG AGA C
Reverse	AGA GAC TCC CCA CAC AGC CA
Probe	CCG GAC GGC CAA GAT GAT CAC C
*sst*_*3*_	
Forward	CTG GGT AAC TCG CTG GTC ATC TA
Reverse	AGC GCC AGG TTG AGG CTG TA
Probe	CGG CCA GCC CTT CAG TCA CCA AC
*sst*_*5*_	
Forward	CAT CCT CTC CTA CGC CAA CAG
Reverse	GGA AGC TCT GGC GGA AGT T
Probe	CCC GTC CTC TAC GGC TTC CTC TCT GA

Pyrosequencing primers	sequence
PCR	
Forward	[Btn]GGG TTG GTT GGG TTA GTT TT
Reverse	ATT CCT AAC TCC TCC ACC CTC TT
Sequencing	
Reverse strand	ACC TCA AAC TAA AAC TCT A

ChIP primers	sequence
-2	
Forward	TGC TGA CTG ACG TGG CTA CA
Reverse	CGC ACC TGG AGT CCA AGA TT
-1	
Forward	GTC CTT GCC ATG AGT CTT GA
Reverse	CAG GCA GAG CTT ACA GAC AG
tss	
Forward	AGC GAA GCC GCT GTG ACG TA
Reverse	TCT GGG CGC TGG TGG TCT TG

### Somatostatin receptor uptake

SSTR internalization studies were performed as previously described with minor adjustments [33]. In short, cells of either cell line, BON-1 and QGP-1, were seeded in 75 cm^2^ culture flasks in 10 ml regular culture medium on day 0. On day 1, four conditions were initiated; control, 5-aza-dC (BON-1: EC_50_ 100 nM, QGP-1: EC_50_ 50 nM); VPA (BON-1: EC_50_ 2.5 mM, QGP-1: EC_50_ 1 mM); and combined 5-aza-dC and VPA treatment. Supplemental medium and compounds were added on day 3. On day 6, the cells are trypsinized and plated in 12 well plates (1 · 10^6^ cells per well), 6 wells per treatment for triplicate incubation with either [^125^I-Tyr^3^]octreotide or [^125^I-Tyr^3^]octreotide and excess unlabeled octreotide to block internalization. On day 8, cells were washed twice with internalization medium (DMEM supplemented with HEPES (30 mM) (Sigma-Aldrich)), L-Glutamine (2 mM), sodium pyruvate (1 mM) (Gibco), penicillin (10^5^ U/L), fungizone (0.5 mg/L) and 0.2% BSA (Sigma-Aldrich) (pH 7.4). Subsequently, 1 ml of internalization medium per well was added to the cells, as well as 200,000 counts [^125^I-Tyr^3^]octreotide (final concentration ∼0.1 nM) was added to the wells and the plates were incubated at 37°C for 4 hours with or without excess unlabeled octreotide. After incubation the cells were washed twice with ice-chilled internalization medium. Low pH treatment with 1 ml sodium acetate (20 mM) (Sigma-Aldrich) in Hanks Balanced salt solution (Gibco), pH 5.0 (HBSS-Ac) was used to collect the membrane bound fraction of [^125^I-Tyr^3^]octreotide. The cells were incubated for 10 minutes at 37°C, subsequently the supernatant was collected (membrane fraction 1) and cells were washed one additional time with HBSS-Ac and supernatant collected (membrane fraction 2, membrane fraction data not shown). Finally, the cells were extracted with 1 M NaOH, representing the internalized fraction of radioactivity. By Riastar (Perkin Elmer) internalized counts per million were determined in each treatment condition and fraction.

### Effect on cAMP production

Cells were plated in 75 cm^2^ culture flasks and left to adhere for 2 days. Subsequently the cells were pretreated with no compound (vehicle); 5-aza-dC; (BON-1: EC_50_ 100 nM, QGP-1: EC_50_ 50 nM); VPA (BON-1: EC_50_ 2.5 mM, QGP-1: EC_50_ 1 mM); or 5-aza-dC and VPA combination for 5 days. After 5 days of incubation with drugs, the cells were trypsinized and plated in a 24 well plate (0.1 · 10^6^ cells per well) without or with compounds for an additional 2 days. After 2 days (total drug treatment period 7 days), the cells were incubated in triplicate without or with forskolin (10^-6^ M; Sigma-Aldrich) or with forskolin plus increasing concentrations of octreotide (range 10^-6^-10^-10^ M; Sandostatin, Novartis, Arnhem, the Netherlands) for 30 minutes in a CO_2_ incubator at 37°C. The supernatant of the cells was collected, snap frozen on dry ice and stored at -80°C. cAMP levels were measured with RIA (Beckman Coulter, Woerden, the Netherlands) according to manufacturer’s protocol.

### DNA isolation, bisulfite treatment and pyrosequencing

DNA was isolated from untreated- and 5-aza-dC; (BON-1: EC_50_ 100 nM, QGP-1: EC_50_ 50 nM) treated BON-1 and QGP-1 cells, according to protocol with the Genome Wizard DNA isolation kit (Promega Corporation, Madison, USA). For bisulfite conversion 1000 ng input DNA was used with the Zymo Research EZ DNA Zymo kit according to manufacturer’s protocol (Zymo Research Corporation, Irvine, USA). Primer design was done with PyroMark Assay Design 2.0 (Qiagen N.V., Venlo, the Netherlands). Bisulfite treated DNA was aliquoted and stored at -20°C. PCR of bisulfite treated DNA was performed with the primers listed in Table 1 and with the following program: 5 minutes 95°C, 45 cycles 30 seconds 95°C - 30 seconds 60°C – 30 seconds 72°C, 1 cycle 10 minutes 72°C, ∞ 4°C. PCR products were analyzed on the PyroMark Q24 (Qiagen) with PyroMark Gold Q24 reagents (Qiagen) according to manufacturer’s protocol

### Chromatin isolation

Chromatin isolation was performed to evaluate H3K9Ac and H3K27me3 enrichment. BON-1 and QGP-1 cells were plated on day 1 and grown until day 4, after which the medium was changed and 5-aza-dC (BON-1 EC_50_ 100 nM, QGP-1 EC_50_ 50 nM); VPA (BON-1 EC_50_ 2.5 mM, QGP-1 EC_50_ 1 mM); and the combination of both drugs were added, except to the control flasks. The cells were incubated for 4 days and harvested on day 8. The cells were trypsinized and centrifuged to remove the trypsin. Cells from the same treatment condition were resuspended and pooled in 15 ml fresh medium in a 50 ml tube. Formaldehyde was added to a final concentration of 1% to cross-link, and incubated rotating at room temperature for 10 minutes. Subsequently 2.5 M glycine was added to a final concentration of 0.125 M to quench the reaction. The fixed cells were then centrifuged at 4°C, 5 minutes at 1500 rpm and washed twice with ice-cold PBS. The cells were resuspended in 750 μl PBS and transferred to a 1.5 ml tube and centrifuged at 4°C for 5 minutes at 3000 rpm. Cells were incubated 10 minutes in 500 μl nucleic lysis buffer (1% SDS + 50 mM Tris-HCL (pH8.1) + 10 mM EDTA (pH8.0) with PMSF on ice. Following incubation the lysates were sheared (cooled on ice), with a mean of 7 times 20 second pulses with 60 second pauses to cool. Samples were centrifuged at 4°C at 13,000 rpm. Shearing is performed to create chromatin fragments of 200-1000bp, which was confirmed by gel electrophoresis following DNA isolation of 50 μl of the 500 μl sample with 30 minutes incubation with 100 μl H_2_O, 6 μl 5 M NaCl and 2 μl RNAse A (10 mg/ml) at 37°C and a subsequent 2 hour incubation with adding of 2 μl proteinase K (20 mg/ml) at 65°C. The DNA was purified with the Roche high pure PCR purification kit.

### Chromatin immunoprecipitation

Prior to precipitation, chromatin was precleared with negative control IgG from rabbit serum for two hours, followed by incubation with protein-G magnetic beads (Invitrogen Dynal AS) for 1 hour, after which the beads were removed and discarded. A 10 μl input sample was set aside and ChIP was performed overnight with 2.5 mg H3K9Ac (ab4441, Abcam, Cambridge, UK) or H3K27me3 (ab6002, Abcam) antibodies, as well as a negative control IgG from rabbit serum, followed by 2 hours incubation with protein-G magnetic beads. Subsequently the beads are sequestered by a magnet and the supernatant removed. The beads were washed 3 times with a low salt buffer (20 mM Tris-HCl (pH 8.0) + 2 mM EDTA + 1 % Triton X-100 + 150 mM NaCl), 1 time a high salt wash (20 mM Tris-HCl (pH 8.0) + 2 mM EDTA + 1 % Triton X-100 + 0.1% SDS + 500 mM NaCl) and 1 time a LiCl wash (10 mM Tris-HCl (pH 8.0) + 1 mM EDTA + 0.25M LiCl + 0.5% IGEPAL + 0.5% Deoxycholate (sodium salt)) for 5 minutes at 4°C. After a subsequent TE buffer wash, 150 μl elution buffer (25 mM Tris-HCl (pH 7.5) + 10 mM EDTA + 0.5% SDS) was added to the beads as well as the input sample, the beads were incubated at 65°C for 30 minutes. The eluted chromatin was then transferred to clean Eppendorf tubes and together with the input samples incubated overnight with 6 μl 5 M NaCl and 2 μl Proteinase K (20 mg/ml). The precipitated DNA and input DNA was isolated by Roche high pure PCR purification kit and eluted in 50 μl H2O.

Enrichment of the *sst*_*2*_ promoter was analyzed at three positions, primer sequences are described in Table 1. Furthermore negative and positive controls were analyzed for both histone marks (data not shown). Q-PCR was performed with Roche Fast start Universal SYBR green master mix (Rox) according to protocol. The percentage recovery was calculated by 100% × E^^(CTinput-adjusted – CtIP)^, with E being the efficiency coefficient of the primers. The correction factor for the adjusted CT for input DNA was calculated by E^^*X*^ =50, with *X* being the correction factor. For the primer sets -2, -1 and tss, the efficiency factors were 1.9, 2.2, 1.9, respectively.

### Statistics

In order to stabilize the variance, the data was log transformed before statistical analysis. For the comparison of treatment means, the Tukey post hoc test method was applied. All data were analyzed using GraphPad (Prism 5, La Jolla, CA, USA). P values <0.05 were considered significant. To compare the cAMP data, we performed an F-test.

## SUPPLEMENTARY MATERIALS FIGURE


