# Supplementary material for: Epidrug-induced upregulation of functional somatostatin type 2 receptors in human pancreatic neuroendocrine tumor cells
Source: Oncotarget. 2016 May 19;9(19):14791–802. doi: 10.18632/oncotarget.9462 (PMC5871078; doi:10.18632/oncotarget.9462)
Supplement: Supplementary file 1 [file oncotarget-09-14791-s001.pdf]

SUPPLEMENTARY FIGURE

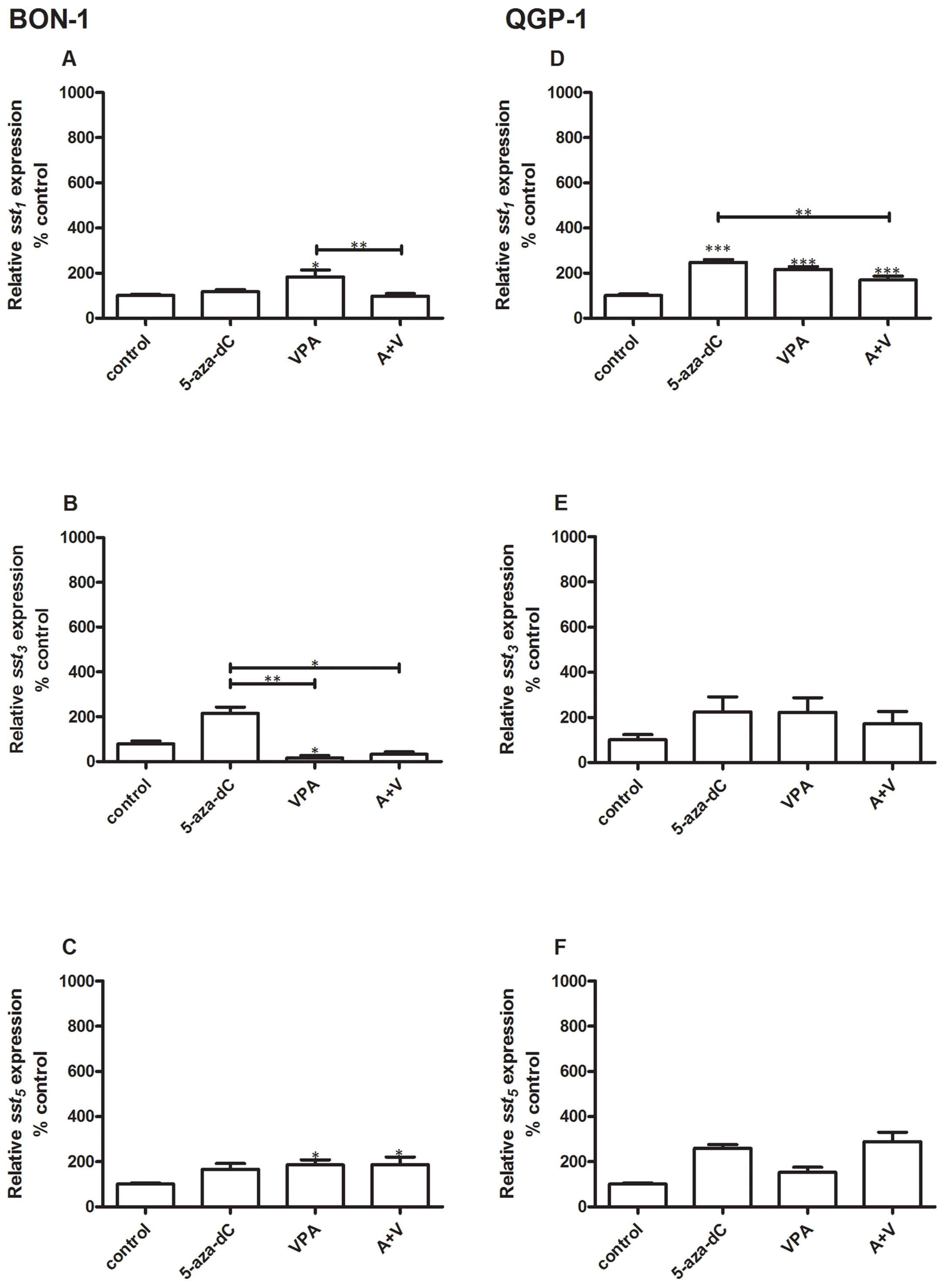

Supplementary Figure 1: A, B, C. effect of treatment with 5-aza-dC (100 nM), VPA (2.5 mM) or the combination of both drugs on sst_1, sst_3 and sst_5 expression in BON-1 cells. **D, E, F.** effect of treatment with 5-aza-dC (50 nM), VPA (1 mM) or the combination of both drugs on sst_1, sst_3 and sst_5 expression in QGP-1 cells. Data are the mean ± SEM and expressed as the percentage of untreated control cells. The expression levels were normalized to housekeeping gene *hprt*. *p<0.05, **p<0.01, ***p<0.001.
